# Challenges in the Management of Cavernoma-Related Epilepsy: Seizure Outcomes, Antiseizure Medication Practices, and Access to Intraoperative Technologies in Kazakhstan

**DOI:** 10.3390/brainsci15090992

**Published:** 2025-09-15

**Authors:** Karashash Menlibayeva, Chingiz Nurimanov, Iroda Mammadinova, Ainur Turzhanova, Serik Akshulakov, Yerbol Makhambetov

**Affiliations:** 1 Department of Population Health Sciences, Faculty of Life Sciences and Medicine, King’s College London, London SE1 1UL, UK; karashash.2.menlibayeva@kcl.ac.uk; 2 Vascular and Functional Neurosurgery Department, National Centre for Neurosurgery, Astana 010000, Kazakhstan; irodamammadinova@gmail.com (I.M.); yermakh@gmail.com (Y.M.); 3 Department of Research Management, National Centre for Neurosurgery, Astana 010000, Kazakhstan

**Keywords:** cerebral cavernous malformations, seizures, ILAE, microsurgical resection, epilepsy

## Abstract

Objective: This study aims to analyze the diagnostic patterns of cavernoma-related epilepsy, the management of antiseizure medications, and clinical outcomes following microsurgical treatment in patients with late-diagnosed epilepsy secondary to cavernous malformations in the Central Asian region. Methods: A retrospective cross-sectional study was conducted on 60 patients who underwent microsurgical resection for brain cavernous malformations over a 12-year period (2010–2022) at the National Centre for Neurosurgery, Astana, Kazakhstan. All participants were 18 years or older and presented with seizures. Follow-up evaluations were conducted by neurologists, and seizure outcomes were assessed using the 2017 classification criteria of the International League Against Epilepsy. Results: The mean follow-up period was 83.77 ± 39.81 months. In total, 51.67% of participants demonstrated positive ILAE outcomes, 33.33% had moderate ILAE outcomes, and the remaining 15.00% experienced negative ILAE outcomes. Approximately 47% of patients received antiseizure medication before surgery, primarily as monotherapy with carbamazepine (33%), and administered at a low dose (40%). Early microsurgical resection showed a positive post-surgery seizure outcome. Approximately 67% of patients who experienced seizures within one year prior to surgery showed positive ILAE outcomes, whereas those with a seizure history extending beyond five years were roughly 32% seizure-free (*p* = 0.01). Conclusions. Cavernoma-related epilepsy in Central Asia remains a significant clinical challenge, particularly with respect to diagnostic accuracy and antiseizure medication management. In our cohort, only approximately half of patients achieved favorable seizure control following microsurgical resection. Notably, early surgical intervention within one year of seizure onset was associated with improved outcomes, whereas delayed surgery, restricted availability of intraoperative technologies, and suboptimal antiseizure medication practices were linked to less favorable outcomes. Strengthening diagnostic pathways, antiseizure medication management, and expanding access to advanced surgical technologies are critical steps to improving treatment outcomes in a studied patient population.

## 1. Introduction

CMs are benign vascular lesions that occur within the brain parenchyma or leptomeninges [1]. Histologically, they are characterized by clusters of dilated vascular caverns lined by endothelium, lacking mature vascular architecture. Although many CMs remain clinically silent, they may present symptomatically, most commonly with intracerebral hemorrhage or seizures [2,3].

Several risk factors are associated with the occurrence of seizures in patients with CMs. These include temporal lobe involvement, cortical location, presence of a hemosiderin rim, patient age, and specific morphological characteristics of the lesion [4,5,6,7]. If left untreated, CM-related seizures may evolve into CRE, the management of which involves pharmacological therapy, microsurgical resection, and, in selected cases, stereotactic radiosurgery [8].

The primary treatment for CM-related seizures involves ASMs, with early initiation being critical given the high risk of epilepsy development after a first seizure—estimated at up to 94% within five years [9]. Among therapeutic options, microsurgical resection has demonstrated superior efficacy compared with medication alone or radiosurgery [8,10]. Evidence indicates that resection achieves favorable outcomes even in patients with drug-resistant CRE [11]. Patient-reported outcomes further support its effectiveness, showing significant long-term seizure control and improved quality of life following surgery [12]. Importantly, early intervention, particularly within one year of symptom onset, is associated with the most favorable outcomes [4,13,14]. Patients undergoing surgical resection frequently attain seizure freedom and may discontinue ASM therapy postoperatively [15]. By contrast, delayed surgery and prolonged seizure duration are associated with poorer outcomes, emphasizing the need for early diagnosis and timely surgical management of CRE [16,17].

The diagnosis of CRE requires a multidisciplinary approach involving neuroradiologists, neurologists, and neurosurgeons. MRI and EEG remain essential for identifying epilepsy of structural etiology [18]. Nevertheless, timely diagnosis continues to be a major challenge, particularly in resource-limited settings. Approximately 80% of epilepsy cases occur in low- and middle-income countries, where access to appropriate diagnostic tools and treatment remains limited [19,20]. Furthermore, societal stigma associated with epilepsy often contributes to delays in seeking medical care [21,22], despite existing World Health Organization guidelines aimed at addressing these challenges [23].

In Kazakhstan, the largest upper-middle-income country in Central Asia [24], epilepsy is frequently undiagnosed or diagnosed late, with rising incidence and prevalence observed in the years preceding the COVID-19 pandemic [25]. Data on seizure outcomes following microsurgical resection of CRE in patients with delayed CM diagnosis remain scarce, and information on ASM management patterns in this context is limited. This study therefore aims to analyze the diagnostic pathways, ASM management practices, and seizure outcomes following microsurgical treatment of CRE in patients with late-diagnosed epilepsy secondary to CM in Kazakhstan.

## 2. Materials and Methods

### 2.1. Study Design

Retrospective cross-sectional study.

### 2.2. Participants

A total of 116 medical records of patients who underwent microsurgical resection for CRE were reviewed to assess eligibility. Of these, 56 patients (48.28%) were excluded from the analysis. Nineteen were lost to follow-up despite repeated contact attempts, thirty-one had not experienced preoperative seizures, and six had died, with the cause of death remaining unknown. Notably, four of these deaths occurred in 2020 during the COVID-19 pandemic. The final study cohort consisted of 60 adult patients who underwent microsurgical resection between 2010 and 2022 at the National Centre for Neurosurgery in Astana, Kazakhstan. In this cohort, the primary indication for surgery was drug-resistant epilepsy. A minority of patients presented with preoperative intracerebral hemorrhage, and surgery was performed electively rather than during the acute phase.

### 2.3. Inclusion and Exclusion Criteria

Adult patients who underwent microsurgical resection for brain CM and presented with CM-related intractable seizures were eligible for inclusion. Only patients who underwent preoperative video-electroencephalographic monitoring to confirm seizure onset localization were included. All patients also underwent preoperative MRI, including T1, T2, and susceptibility-weighted imaging sequences, to characterize the lesion and assess for evidence of hemorrhage.

Patients younger than 18 years, those with incomplete or lost follow-up data, and those with multiple cavernous malformations were excluded to reduce heterogeneity and minimize potential confounding factors influencing seizure outcomes.

### 2.4. Follow-Up

Neurologists contacted patients by phone and conducted interviews. Follow-up data were subsequently evaluated by an epileptologist and categorized according to the ILAE classifications of epilepsy surgery seizure outcomes. In this study, ILAE classes 1 and 2 were defined as positive outcomes, classes 3 and 4 as moderate outcomes, and classes 5 and 6 as negative outcomes.

During the follow-up period, all patients also underwent postoperative electroencephalographic evaluations. These recordings were reviewed by board-certified epileptologists to detect persistent epileptiform activity and to evaluate the effectiveness of seizure control.

### 2.5. Variables

Data were collected on participants’ demographic and clinical characteristics, CM features, and preoperative as well as postoperative antiseizure medication use. The place of residence was categorized as either a metropolis or a small city. In Kazakhstan, three cities—Astana (the capital), Almaty, and Shymkent—are officially classified as metropolises, each with a population exceeding one million inhabitants. All other towns were grouped as small cities.

The variable “other nationalities” included Russians, Uzbeks, Germans, and Tatars. Seizure types were classified according to the ILAE 2017 Classification of Seizure Types and were further grouped into focal onset, generalized onset, and unknown onset.

Comorbidities were categorized into cardiovascular diseases (arterial hypertension, congestive heart failure, coronary disease, and angina), gastrointestinal disorders (chronic pancreatitis, chronic gastritis, chronic cholecystitis, peptic ulcer, and hepatitis B and C), renal diseases (chronic pyelonephritis, chronic renal disease, malignant neoplasm of the kidney, chronic glomerulonephritis, and nephrolithiasis), respiratory system diseases (chronic bronchitis and pulmonary fibrosis), endocrine system diseases (Huntington’s disease, multinodular goiter, immunologic thyroiditis, type 2 diabetes, and cardiometabolic syndrome), and ophthalmological conditions (retinal angiopathy, cataract, astigmatism, and manifest deviation).

Antiseizure medication dosages were categorized according to the maintenance dosage defined in the epilepsy treatment protocol of the Ministry of Health of Kazakhstan. A dosage below the maintenance level was categorized as low, while a dosage above the maintenance level was categorized as high. The following maintenance dose ranges were used: carbamazepine 600–1200 mg/day, sodium valproate 1000–3000 mg/day, valproic acid 1000–3000 mg/day, levetiracetam 1000–3000 mg/day, lamotrigine 100–200 mg/day, oxcarbazepine 900–2400 mg/day, and topiramate 200–400 mg/day [26].

### 2.6. Statistical Methods

Data were cleaned and coded using Microsoft Excel (version 2411, Microsoft 365). Further statistical analysis was performed in Stata 18.0 SE (StataCorp, College Station, TX, USA). Chi-square and Fisher’s exact tests were used, as appropriate, to assess associations between categorical variables. Continuous variables were assessed for normality using the skewness–kurtosis test and visualized using histograms. As the data were not normally distributed, the Kruskal–Wallis test was employed to compare continuous variables across ILAE outcome groups.

## 3. Results

A total of 60 patients with complete follow-up data were included in the final analysis. The mean follow-up duration was 83.77 ± 39.81 months. The mean age at surgery was 36.98 ± 11.78 years, while the mean age at initial radiographic diagnosis (MRI/CT) was 35.90 ± 11.16 years, and the mean age at first seizure was 32.95 ± 12.22 years.

Most participants were of Kazakhs ethnicity (76.67%) and male (58.33%), with the majority residing in small cities (76.67%). Overall, a tenth of patients reported having a disability, and 15% had experienced a preoperative intracerebral hemorrhage. Headache was the most frequent initial symptom (85.00%), followed by weakness, numbness, or paralysis (38.33%), memory impairment (16.67%), and hearing or vision disturbances with unsteadiness (10.00%).

All patients (100%) presented with seizures. The mean duration of seizures prior to surgery was 48.45 ± 54.65 months, whereas the mean time interval between radiographic diagnosis of CM and surgery was 11.91 ± 31.17 months.

Most participants experienced generalized seizures (40.00%), while 19 patients (31.67%) presented with focal-onset seizures, and 17 patients (28.33%) had seizures of unknown onset. The most prevalent comorbidity was cardiovascular disease (15.00%), including arterial hypertension, congestive heart failure, coronary artery disease, and angina.

Regarding seizure location, approximately one-third of CMs were discovered in the frontal lobe, 28.33% in the temporal lobe, one-fifth in the parietal lobe, 12.00% in deep brain structures, and 5.00% in the occipital lobe. Laterality analysis showed that 53.33% of lesions were located in the left hemisphere.

The demographic and clinical characteristics of the cohort are summarized in Table 1.

Table 2 summarizes the preoperative (Preop) and postoperative (Postop) use of antiseizure medications (ASMs), including dosage patterns. More than half of participants (53.33%) did not receive any ASM prior to surgery. Among those treated, carbamazepine was the most frequently prescribed agent, both before and after resection. Overall, prescribed dosages tended to be at the lower end of the recommended therapeutic range.

The relationship between patients’ antiseizure medication (ASM) use and their ILAE seizure outcomes is presented in Table 3.

Among the 60 patients, 28 (46.67%) received ASM therapy prior to surgery, and 25 of this continued treatment postoperatively. Among those who continued ASM, 56% achieved positive ILAE outcomes at long-term follow-up. By contrast, 32 patients (53.33%) did not receive medication before surgery, with half of them initiating treatment postoperatively. Among those, 56.25% achieved positive long-term outcomes. However, the differences between groups were not statistically significant. The role of preoperative ASM therapy is further analyzed and presented in Table 4.

Among patients who received ASMs prior to surgery (N = 28), treatment with carbamazepine at initial dosing levels was associated with higher rate of favorable ILAE outcomes, with 53.57% achieving seizure control.

The seizure outcomes following microsurgical resection of CM in the entire cohort of 60 patients are summarized in Table 5. The mean follow-up duration was 83.77 ± 39.81 months (range, 14–169 months). At the final follow-up, 51.67% of patients achieved favorable outcomes, 33.33% had moderate outcomes, and the remaining 15.00% experienced negative ILAE outcomes.

The mean age at surgery was 37 years, with the youngest subgroup observed among patients with moderate ILAE outcomes (33.65 ± 8.08 years). Among patients with a history of intracerebral hemorrhage, 71.43% achieved moderate or favorable outcomes, compared with 86.8% of those without hemorrhage. CMs located in deep brain structures were predominantly associated with moderate outcomes (57.14%), whereas lesions in other brain regions more frequently resulted in favorable outcomes.

The only statistically significant association was found for seizure duration prior to surgery. Approximately 67% of patients who underwent surgery within one year of seizure onset achieved favorable outcomes, whereas only 32% of those with a seizure history extending five years attained favorable outcomes.

## 4. Discussion

This study evaluated diagnostic patterns of CREs, the management of ASMs, and postoperative seizure outcomes in patients with late-diagnosed epilepsy secondary to cerebral CM in a Kazakhstani cohort. Overall, 51.67% of patients achieved favorable long-term outcomes (ILAE classes 1–2) after surgery, with a mean follow-up of approximately seven years. These results were less favorable compared to prior studies, which have reported seizure freedom rates of about 70% following CM resection [17,27].

A deeper understanding of the mechanisms of epileptogenesis in CM is critical for interpreting surgical outcomes. Although CMs themselves are not intrinsically epileptogenic, epilepsy arises through two primary mechanisms: (1) local epileptogenesis of the surrounding tissue and (2) secondary epileptogenesis in remote brain regions [28]. Cortical excitability surrounding CMs, driven by reactive gliosis, hemosiderin deposits, and architectural disturbances, plays a key role in the development of CRE [29]. In addition, secondary epileptogenesis in distant brain areas underscores the intricate network changes and synaptic alterations that perpetuate seizure activity, particularly within the limbic system. A comprehensive understanding of these processes will inform more targeted therapeutic strategies aimed at improving seizure control in patients with CMs [28].

Studies from other countries provide valuable context for interpreting our findings on CRE. Microsurgical resection remains the most effective treatment for seizure control, particularly for lesions in the temporal and frontal lobes, achieving higher rates of epilepsy control compared to radiosurgery, although neurosurgery may carry a slightly higher risk of permanent morbidity [8,17].

Emerging minimally invasive techniques, such as laser interstitial thermal therapy (LITT), have shown promise for seizure control and prevention of symptomatic progression or hemorrhage in selected patients [30]. For patients with lesions considered unsuitable for surgery due to location or other risk factors, stereotactic radiosurgery offers an alternative, providing meaningful reductions in bleeding and seizure frequency with relatively low adverse effects [31].

Advanced surgical techniques, such as 3.0 Tesla MRI with an epilepsy-specific protocol, intraoperative electrocorticography, modern navigation systems, and cortical mapping, are critical for achieving seizure freedom after surgery [32,33]. Because the number and localization of lesions strongly influence post-surgery outcomes [8], these innovations, together with the expertise of trained epileptologists, have substantially improved surgical outcomes by enabling more precise identification and resection of epileptogenic tissue [34,35,36].

In the present cohort, the limited availability of advanced intraoperative technologies—introduced at our center only in 2021—likely contributed to suboptimal resection accuracy and the relatively modest seizure-free rate observed (51.67%). The absence of intraoperative MRI or CT, which can delineate the hemosiderin rim and facilitate more complete lesion removal, further limited surgical efficacy. These limitations represented significant barriers to optimal epilepsy surgery outcomes in Kazakhstan until recently. Looking ahead, we plan to conduct a comparative study assessing seizure outcomes in CM patients operated on with intraoperative EEG and MRI versus those treated without these tools.

Lesion location is a critical determinant of both the choice of intervention and the likelihood of favorable surgical outcomes. Temporal CMs, particularly those in the mesiotemporal lobe, are strongly associated with the development of CRE, as this region represents the most epileptogenic zone [37]. For CMs located in the frontal and temporal lobes, microsurgical resection generally offers superior seizure control compared with radiosurgery. By contrast, radiosurgery has demonstrated greater effectiveness in managing CMs situated in the parietal and occipital lobes [38]. In the present cohort, however, no differences were observed between the CM location and the post-surgery seizure outcome.

Another key factor associated with positive post-surgery seizure freedom is the duration of epilepsy prior to surgery [14]. In this study, the interval between the initial presenting seizure and surgery demonstrated a statistically significant association with postoperative ILAE outcomes. Prolonged seizure duration before surgery was associated with poorer outcomes, whereas patients whose first seizure occurred within one year prior to surgery achieved substantially better results, with approximately 67% achieving seizure freedom (ILAE classes 1–2).

These findings were consistent with prior studies. Early surgical intervention has been shown to be effective in multiple settings, including a cohort from Sheffield [39] and large-volume cerebrovascular centers [14,40]. However, those studies largely evaluated outcomes of microsurgery following the initial seizure without explicitly considering the timing of CM diagnosis as the underlying etiology. In the present cohort, delayed surgical intervention was primarily attributable to late diagnosis of CM rather than to clinical decision making alone. These results reinforce existing evidence supporting early microsurgical intervention for seizure control in CRE, while also highlighting the importance of timely and accurate diagnosis of CM to optimize surgical outcomes.

In the analyzed cohort, a substantial delay was observed between the onset of initial seizures and the confirmation of a diagnosis of CM and associated epilepsy using diagnostic tools such as MRI, CT, or EEG. The number of patients who underwent microsurgery within one year after radiographic confirmation of CRE was approximately twice that of patients who pursued surgical intervention after the initial seizure (54 vs. 27). The reasons for this delay remain unclear but may be related to limited access to primary care services or to the strong stigma associated with epilepsy [41,42]. As reported by Guo and colleagues, patients with epilepsy often conceal their condition and withdraw from social interactions [41]. This behavior appears to be particularly common in Asian countries [43,44], where self-perception of the diagnosis and coping mechanisms exert greater influence on psychosocial adjustment than the clinical manifestations of epilepsy themselves [45]. Moreover, negative societal attitudes toward marriage, parenthood, and employment are more prevalent in culturally homogeneous societies [46,47]. By contrast, public perceptions in Western countries are generally more favorable [48,49], with only about 10% of respondents expressing negative attitudes toward people with epilepsy [50], and these views have remained relatively stable for nearly two decades [51]. In the studied Kazakhstani cohort, epilepsy-related stigma may have contributed to diagnostic delays, late surgical intervention, and, consequently, less favorable postoperative outcomes.

Another critical aspect concerns the management of ASMs in Kazakhstan. Inappropriate prescribing practices remain a significant challenge, with monotherapy being the most common strategy [52]. Suboptimal medication selection and underdosing likely contribute to inadequate seizure control both before and after surgery [53]. In this study cohort, only 46.67% of patients with CRE received ASM therapy, most commonly low-dose carbamazepine monotherapy. Similar findings were reported by Guekht and colleagues, who observed that nearly one-quarter of individuals with epilepsy were not receiving pharmacological treatment and that prescribed regimens were frequently suboptimal [54]. This predominance of low-dose monotherapy in our cohort raises concerns regarding limited adherence to treatment protocols, inappropriate prescribing practices, and possible restrictions in ASM availability within the country.

### 4.1. Limitations

This study has several limitations. First, its retrospective design constrained the scope and accuracy of data collection. Reliance on self-reported outcomes may have introduced self-selection and survivorship bias.

Second, the proportion of patients lost to follow-up may have influenced the results. Several context-specific factors likely contributed to this attrition, including inadequate medical record management, underdeveloped digital medical record infrastructure resulting in loss of contact information, and cultural attitudes toward health disclosure. In some cases, patients expressed reluctance or skepticism in responding to health-related questions, even when contacted by their treating physicians.

Third, the relatively small sample size may have limited the statistical power of the study and reduced the generalizability of the findings. Finally, the lack of clinical data regarding the resection of hemosiderin-stained areas restricted the analysis. The absence of intraoperative imaging and navigation systems impeded consistent identification and documentation of these regions, thereby preventing a thorough evaluation of their association with CRE.

### 4.2. Future Directions

Further research is warranted to investigate the patterns of diagnosis and management of patients presenting with seizures, as well as the practices of physicians in prescribing diagnostic tests following an initial seizure episode. Additionally, a survey studying attitudes toward epilepsy, as well as self-stigma among patients, are needed to better understand barriers to timely care. Studies evaluating patterns of ASM prescribing and administration would also provide valuable insights into current treatment gaps. Moreover, a comparative analysis of postoperative seizure outcomes in patients with CRE before and after the implementation of intraoperative tools is essential, alongside efforts to address persistent limitations in early diagnosis and treatment approaches.

## 5. Conclusions

Seizure control following microsurgical resection of CRE in Kazakhstan was limited, with favorable outcomes achieved in only half of patients. Early surgery within one year of seizure onset was associated with improved outcomes, whereas delayed surgery, restricted access to intraoperative resources, and suboptimal ASM management were associated with less favorable outcomes. Strengthening diagnostic pathways, optimizing ASM management, and advancing surgical technology are essential steps toward improving outcomes in this patient population.

## Figures and Tables

**Table 1:** Baseline demographic and clinical characteristics of 60 patients.

Variable	n (%)
No. of patients	60 (100)
Mean follow-up time (months) ± SD	83.77 ± 39.81
Age (years) at the time of surgery	
Mean ± SD	36.98 ± 11.78
16–30	24 (40.00)
31–40	14 (23.33)
41 and older	22 (36.67)
Age (years) at first radiographic diagnosis (MRI/CT) of CM	
Mean ± SD	35.90 ± 11.16
16–30	26 (43.33)
31–40	14 (23.33)
41 and older	20 (33.33)
Age (years) when the initial seizure occurs	
Mean ± SD	32.95 ± 12.22
16–30	27 (45.00)
31–40	19 (31.67)
41 and older	14 (23.33)
Gender	
Female	25 (41.67)
Male	35 (58.33)
Nationality	
Kazakh	46 (76.67)
Other	14 (23.33)
City type	
Metropolises	14 (23.33)
Small cities	46 (76.67)
Disability	
Yes	6 (10.00)
No	54 (90.00)
Time since radiographic diagnosis (months)	
Mean ± SD	11.91 ± 31.17
<1 year	54 (90.00)
1–5 year	1 (1.67)
>5 years	5 (8.33)
Time since initial presenting seizure (months)	
Mean ± SD	48.45 ± 54.65
<1 year	27 (45.00)
1–5 year	14 (23.33)
>5 years	19 (31.67)
Type of seizure	
Focal onset	19 (31.67)
Generalized onset	24 (40.00)
Unknown onset	17 (28.33)
Preoperative hemorrhage	
Yes	9 (15.00)
No	51 (85.00)
Initial presenting symptoms	
Headaches	51 (85.00)
Hearing or vision changes	6 (10.00)
Weakness, numbness, or paralysis	23 (38.33)
Memory deficits	10 (16.67)
Unsteadiness	6 (10.00)
Comorbidities	
Cardiovascular system diseases	9 (15.00)
Gastrointestinal system diseases	5 (8.33)
Renal system diseases	3 (5.00)
Respiratory system diseases	1. (1.67)
Endocrine system diseases	3 (5.00)
Ophthalmological condition	2 (3.33)
Location of the cavernous malformation	
Deep brain structures	7 (11.67)
Frontal lobe	21 (35.00)
Occipital lobe	3 (5.00)
Parietal lobe	12 (20.00)
Temporal lobe	17 (28.33)
Location of the cavernous malformation (side)	
Left	32 (53.33)
Right	28 (46.67)

**Table 2:** Preoperative and postoperative data on ASM and ILAE outcomes.

Variable	Preop, n (%)	Postop, n (%)
Treatment with ASM		
Yes	28 (46.67)	41 (68.33)
No	32 (53.33)	19 (31.67)
Medication Name		
Carbamazepine	20 (33.33)	31 (51.67)
Lamotrigine	2 (3.33)	1 (1.67)
Topiramate	1 (1.67)	1 (1.67)
Valproic acid	5 (8.33)	8 (13.33)
None	32 (53.33)	19 (31.67)
Medication Dosage		
Initial dose	24 (40.00)	38 (63.33)
Maintenance dose	4 (6.67)	3 (5.00)
None	32 (53.33)	19 (31.67)

**Table 3:** Impact of medication behavior on ILAE outcomes.

Variable, n (%)	Negative ILAE Outcomes	Moderate ILAE Outcomes	Positive ILAE Outcomes	All
No. of patients	9 (15.00)	20 (33.33)	31 (51.67)	60 (100)
Patients who took medication before surgery and continued after surgery	8 (32.00)	3 (12.00)	14 (56.00)	25 (41.67)
Patients who took medication before surgery and discontinued after surgery	1 (33.33)	1 (33.33)	1 (33.33)	3 (5.00)
Patients who did not take medication before surgery and did not start after surgery	7 (43.75)	2 (12.50)	7 (43.75)	16 (26.67)
Patients who did not take medication before surgery and started after surgery	4 (25.00)	3 (18.75)	9 (56.25)	16 (26.67)

**Table 4:** Preoperative ASM management on ILAE outcomes.

Variable, n (%)	Negative ILAE Outcomes	Moderate ILAE Outcomes	Positive ILAE Outcomes	All
The number of patients who received preop ASM	9 (32.14)	4 (14.29)	15 (53.57)	28 (100)
Preop ASM name				
Carbamazepine	7 (35.00)	2 (10.00)	11 (55.00)	20 (71.43)
Lamotrigine	0	1 (50.00)	1 (50.00)	2 (7.14)
Topiramate	1 (100.00)	0	0	1 (3.57)
Valproic acid	1 (20.00)	1 (20.00)	3 (60.00)	5 (17.86)
Preop ASM dosage				
Initial dose	9 (37.50)	4 (16.67)	11 (45.83)	24 (85.71)
Maintenance dose	0	0	4 (100)	4 (14.29)

**Table 5:** Seizure outcomes after surgical resection of CM in 60 patients.

Variable, n (%)	Negative ILAE Outcomes	Moderate ILAE Outcomes	Positive ILAE Outcomes	All
No. of patients	9 (15.00)	20 (33.33)	31 (51.67)	60 (100)
Mean follow-up time (months) ± SD	93.11 ± 27.87	83.25 ± 40.22	81.39 ± 43.04	83.77 ± 39.81
Age (years) at the time of surgery, mean ± SD	37.44 ± 10.72	33.65 ± 8.08	39.00 ± 13.75	36.98 ± 11.78
Age (years) at the time of initial seizures, mean ± SD	34.54 ± 11.68	28.02 ± 10.13	35.66 ± 12.94	32.95 ± 12.22
Age (years) at the time of radiographic diagnosis (MRI/CT) of CM, mean ± SD	37.11 ± 10.88	32.90 ± 8.19	37.48 ±12.72	35.90 ± 11.16
Gender				
Female	3 (12.00)	11 (44.00)	11 (44.00)	25 (41.67)
Male	6 (17.14)	9 (25.71)	20 (57.14)	35 (58.33)
Type of seizure				
Focal onset	2 (10.53)	10 (52.63)	7 (36.84)	19 (31.67)
Generalized onset	4 (16.67)	6 (25.00)	14 (58.33)	24 (40.00)
Unknown onset	3 (17.65)	4 (23.53)	10 (58.82)	17 (28.33)
Preoperative hemorrhage				
Yes	2 (28.57)	2 (28.57)	3 (42.86)	7 (11.67)
No	7 (13.21)	18 (33.96)	28 (52.83)	53 (88.33)
Treatment with ASM (preop)				
Yes	4 (14.29)	9 (32.14)	15 (53.57)	28 (46.67)
No	5 (15.62)	11 (34.38)	16 (50.00)	32 (53.33)
Preop ASM dosage				
Low	4 (16.67)	9 (37.50)	11 (45.83)	24 (40.00)
Maintenance	0	0	4 (100.00)	4 (6.67)
None	5 (15.62)	11 (34.38)	16 (50.00)	32 (53.33)
Location of the cavernous malformation				
Deep brain structures	1 (14.29)	4 (57.14)	2 (28.57)	7 (11.67)
Frontal lobe	3 (14.29)	5 (23.81)	13 (61.90)	21 (35.00)
Occipital lobe	0	0	3 (100)	3 (5.00)
Parietal lobe	1 (8.33)	5 (41.67)	6 (50.00)	12 (20.00)
Temporal lobe	9 (15.00)	20 (33.33)	31 (51.67)	17 (28.33)
Location of the cavernous malformation (side)				
Left	3 (9.38)	11 (34.38)	18 (56.25)	32 (53.33)
Right	6 (21.43)	9 (32.14)	13 (46.43)	28 (46.67)
Time since initial presenting seizure *				
**<1 year**	**3 (11.11)**	**6 (22.22)**	**18 (66.67)**	**27 (45.00)**
**1–5 year**	**5 (35.71)**	**2 (14.29)**	**7 (50.00)**	**14 (23.33)**
**>5 years**	**1 (5.26)**	**12 (63.16)**	**6 (31.58)**	**19 (31.67)** (* *p* = 0.01)
Time since radiographic diagnosis				
<1 year	9 (16.67)	18 (33.33)	27 (50.00)	54 (90.00)
1–5 year	0	0	1 (100)	1 (1.67)
>5 years	0	2 (40.00)	3 (60.00)	5 (8.33)

Bold indicates statistically significant (* *p* = 0.01).

## Data Availability

The data presented in this study are available from the corresponding author upon reasonable request. De-identified data may be shared with qualified researchers to ensure the protection of patient confidentiality.

## Abbreviations

CM	Cavernous malformations
CRE	CM-related epilepsy
ASM	Antiseizure medication
MRI	Magnetic Resonance Imaging
EEG	Electroencephalogram
ILAE	International League Against Epilepsy
CT	Computed Tomography

## References

[1] Smith E.R., Scott R.M. (2010). Cavernous Malformations. Neurosurg. Clin. N. Am..

[2] Rosenow F., Alonso-Vanegas M.A., Baumgartner C., Blümcke I., Carreño M., Gizewski E.R., Hamer H.M., Knake S., Kahane P., Lüders H.O. (2013). Cavernoma-Related Epilepsy: Review and Recommendations for Management—Report of the Surgical Task Force of the ILAE Commission on Therapeutic Strategies. Epilepsia.

[3] Alalfi M.O., Lanzino G., Flemming K.D. (2023). Clinical Presentation, Hemorrhage Risk, and Outcome in Patients with Familial Cavernous Malformations: A Pragmatic Prospective Analysis of 75 Patients. J. Neurosurg..

[4] Huang C., Chen M.-W., Si Y., Li J.-M., Zhou D. (2013). Factors Associated with Epileptic Seizure of Cavernous Malformations in the Central Nervous System in West China. Pak. J. Med. Sci..

[5] Zhang P., Zhang H., Shi C., Zhou J., Dong J., Liang M., Li R., Cheng J., Chen Y., Yuan J. (2023). Clinical Characteristics and Risk Factors of Cerebral Cavernous Malformation-Related Epilepsy. Epilepsy Behav..

[6] Menzler K., Chen X., Thiel P., Iwinska-Zelder J., Miller D., Reuss A., Hamer H.M., Reis J., Pagenstecher A., Knake S. (2010). Epileptogenicity of Cavernomas Depends on (Archi-) Cortical Localization. Neurosurgery.

[7] Narita M., Miyairi Y., Motobayashi M., Chiba A., Inaba Y. (2023). Incidence of Cerebral Cavernous Malformation-Related Epilepsy in Children: A Single Center Survey. Cureus.

[8] Gao X., Yue K., Sun J., Cao Y., Zhao B., Zhang H., Dai S., Zhang L., Luo P., Jiang X. (2020). Treatment of Cerebral Cavernous Malformations Presenting with Seizures: A Systematic Review and Meta-Analysis. Front. Neurol..

[9] Josephson C.B., Leach J.-P., Duncan R., Roberts R.C., Counsell C.E., Al-Shahi Salman R. (2011). Seizure Risk from Cavernous or Arteriovenous Malformations. Neurology.

[10] Santos A.N., Rauschenbach L., Riess C., Georgiades I., Fiçilar B., Gallardo E.G., Quesada C.M., Li Y., Tippelt S., Dohna-Schwake C. (2023). Outcome after Conservative or Surgical Treatment for New-Onset Epilepsy in Children with Cerebral Cavernous Malformation. Seizure.

[11] Schuss P., Marx J., Borger V., Brandecker S., Güresir Á., Hadjiathanasiou A., Hamed M., Schneider M., Surges R., Vatter H. (2020). Cavernoma-Related Epilepsy in Cavernous Malformations Located within the Temporal Lobe: Surgical Management and Seizure Outcome. Neurosurg. Focus..

[12] Van Gompel J.J., Marsh W.R., Meyer F.B., Worrell G.A. (2010). Patient-Assessed Satisfaction and Outcome after Microsurgical Resection of Cavernomas Causing Epilepsy. Neurosurg. Focus.

[13] Kapadia M., Walwema M., Smith T.R., Bellinski I., Batjer H., Getch C., Rosenow J.M., Bendok B.R., Schuele S.U. (2021). Seizure Outcome in Patients with Cavernous Malformation after Early Surgery. Epilepsy Behav..

[14] Nico E., Adereti C.O., Hackett A.M., Bianconi A., Naik A., Eberle A.T., Cifre Serra P.J., Koester S.W., Malnik S.L., Fox B.M. (2024). Assessing the Relationship between Surgical Timing and Postoperative Seizure Outcomes in Cavernoma-Related Epilepsy: A Single-Institution Retrospective Analysis of 63 Patients with a Review of the Literature. Brain Sci..

[15] Dammann P., Wrede K., Jabbarli R., Neuschulte S., Menzler K., Zhu Y., Özkan N., Müller O., Forsting M., Rosenow F. (2017). Outcome after Conservative Management or Surgical Treatment for New-Onset Epilepsy in Cerebral Cavernous Malformation. J. Neurosurg..

[16] von der Brelie C., Kuczaty S., von Lehe M. (2014). Surgical Management and Long-Term Outcome of Pediatric Patients with Different Subtypes of Epilepsy Associated with Cerebral Cavernous Malformations. J. Neurosurg. Pediatr..

[17] Shoubash L., Nowak S., Greisert S., Al Menabbawy A., Rathmann E., von Podewils F., Fleck S., Schroeder H.H.W. (2023). Cavernoma-Related Epilepsy: Postoperative Epilepsy Outcome and Analysis of the Predictive Factors, Case Series. World Neurosurg..

[18] Cerebral Angioma. https://www.epilepsydiagnosis.org/aetiology/cerebral-angioma-genetics.html.

[19] Epilepsy: A Public Health Imperative. https://www.who.int/publications/i/item/epilepsy-a-public-health-imperative.

[20] Bankole N.D.A., Dokponou Y.C.H., Koning R.D., Dalle D.U., Kesici Ö., Egu C., Ikwuegbuenyi C., Adegboyega G., Ooi S.Z.Y., Dada O.E. (2024). Epilepsy Care and Outcome in Low- and Middle-Income Countries: A Scoping Review. J. Neurosci. Rural. Pract..

[21] Pellinen J. (2022). Treatment Gaps in Epilepsy. Front. Epidemiol..

[22] Kuehn B. (2019). Fighting Epilepsy Stigma. JAMA.

[23] Katchanov J., Birbeck G.L. (2012). Epilepsy Care Guidelines for Low- and Middle- Income Countries: From WHO Mental Health GAP to National Programs. BMC Med..

[24] World Bank Country and Lending Groups—World Bank Data Help Desk. https://datahelpdesk.worldbank.org/knowledgebase/articles/906519-world-bank-country-and-lending-groups.

[25] Akhmedullin R., Kozhobekova B., Gusmanov A., Aimyshev T., Utebekov Z., Kyrgyzbay G., Shpekov A., Gaipov A. (2024). Epilepsy Trends in Kazakhstan: A Retrospective Longitudinal Study Using Data from Unified National Electronic Health System 2014–2020. Seizure Eur. J. Epilepsy.

[26] Эпилепсия у Детей и Взрoслых > Клинические Прoтoкoлы МЗ РК—2016 (Казахстан) > MedElement. https://diseases.medelement.com/disease/%D1%8D%D0%BF%D0%B8%D0%BB%D0%B5%D0%BF%D1%81%D0%B8%D1%8F-%D1%83-%D0%B4%D0%B5%D1%82%D0%B5%D0%B9-%D0%B8-%D0%B2%D0%B7%D1%80%D0%BE%D1%81%D0%BB%D1%8B%D1%85/14753.

[27] Cossu M., Raneri F., Casaceli G., Gozzo F., Pelliccia V., Lo Russo G. (2015). Surgical Treatment of Cavernoma-Related Epilepsy. J. Neurosurg. Sci..

[28] Jehi L.E., Palmini A., Aryal U., Coras R., Paglioli E. (2014). Cerebral Cavernous Malformations in the Setting of Focal Epilepsies: Pathological Findings, Clinical Characteristics, and Surgical Treatment Principles. Acta Neuropathol..

[29] Rajeswarie R., Aravinda H., Arivazhagan A., Bevinahalli N.N., Rao M.B., Mahadevan A. (2022). Evaluating the Role of Perilesional Tissue in Pathobiology of Epileptogenesis of Vascular Malformations of the Central Nervous System. J. Epilepsy Res..

[30] Jimenez A.E., Geist E.G., Connolly E.S., McKhann G.M., Youngerman B.E. (2025). Laser Interstitial Thermal Therapy for Cavernous Malformations: A Meta-Analysis of Individual Patient-Level Data. J. Neurosurg..

[31] Berber T., Celik S.E., Aksaray F., Yoney A., Harmanci K., Tambas M., Yılmaz B.D., Numanoglu C., Yolcu A., Açan H.İ. (2023). Radiosurgery Effects and Adverse Effects in Symptomatic Eloquent Brain-Located Cavernomas. J. Radiat. Res..

[32] Yang T., Hakimian S., Schwartz T.H. (2014). Intraoperative ElectroCorticoGraphy (ECog): Indications, Techniques, and Utility in Epilepsy Surgery. Epileptic Disord..

[33] Vakani R., Nair D.R. (2019). Electrocorticography and Functional Mapping. Handb. Clin. Neurol..

[34] He K., Alriashy M.H.S., Fan Z., Qiao N., Liao Y., An Q., Xu B., Song J., Zhang X., Zhu W. (2022). Cavernoma-Associated Epilepsy Within the Mesial Temporal Lobe: Surgical Management and Seizure Outcome. World Neurosurg..

[35] Phal P.M., Usmanov A., Nesbit G.M., Anderson J.C., Spencer D., Wang P., Helwig J.A., Roberts C., Hamilton B.E. (2008). Qualitative Comparison of 3-T and 1.5-T MRI in the Evaluation of Epilepsy. AJR Am. J. Roentgenol..

[36] Prat-Acín R., Galeano-Senabre I., López-Ruiz P., García-Sánchez D., Ayuso-Sacido A., Espert-Tortajada R. (2021). Intraoperative Brain Mapping during Awake Surgery in Symptomatic Supratentorial Cavernomas. Neurocirugía.

[37] Shih Y.-C., Chou C.-C., Peng S.-J., Yu H.-Y., Hsu S.P.C., Lin C.-F., Lee C.-C., Yang H.-C., Chen Y.-C., Kwan S.-Y. (2022). Clinical Characteristics and Long-Term Outcome of Cerebral Cavernous Malformations-Related Epilepsy. Epilepsia.

[38] Tuleasca C., Peciu-Florianu I., Strachowski O., Derre B., Vannod-Michel Q., Reyns N. (2023). How to Combine the Use of Intraoperative Magnetic Resonance Imaging (MRI) and Awake Craniotomy for Microsurgical Resection of Hemorrhagic Cavernous Malformation in Eloquent Area: A Case Report. J. Med. Case Rep..

[39] Alexander R., Kirsty H., Imron H., Saminderjit K., Shungu U., Dev B., Varduhi C. (2023). Cavernoma Related Epilepsy—A Sheffield Cohort. J. Neurol. Neurosurg. Psychiatry.

[40] Dziedzic T.A., Koczyk K., Nowak A., Maj E., Marchel A. (2022). Long-Term Management of Seizures after Surgical Treatment of Supratentorial Cavernous Malformations: A Retrospective Single Centre Study. J. Korean Neurosurg. Soc..

[41] Guo W., Wu J., Wang W., Guan B., Snape D., Baker G.A., Jacoby A. (2012). The Stigma of People with Epilepsy Is Demonstrated at the Internalized, Interpersonal and Institutional Level in a Specific Socio-Cultural Context: Findings from an Ethnographic Study in Rural China. Epilepsy Behav..

[42] Tian N., Kobau R., Zack M.M., Greenlund K.J. (2022). Barriers to and Disparities in Access to Health Care Among Adults Aged ≥18 Years with Epilepsy—United States, 2015 and 2017. MMWR Morb. Mortal. Wkly. Rep..

[43] Demirci S., Dönmez C.M., Gündoğar D., Baydar C.L. (2007). Public Awareness of, Attitudes toward, and Understanding of Epilepsy in Isparta, Turkey. Epilepsy Behav..

[44] Lai C.W., Huang X.S., Lai Y.H., Zhang Z.Q., Liu G.J., Yang M.Z. (1990). Survey of Public Awareness, Understanding, and Attitudes toward Epilepsy in Henan Province, China. Epilepsia.

[45] Lau V.W., Lee T.M., Ng P.K., Wong V.C. (2001). Psychosocial Adjustment of People with Epilepsy in Hong Kong. Epilepsia.

[46] Choi-Kwon S., Park K.A., Lee H.J., Park M.S., Lee C.H., Cheon S.E., Youn M.H., Lee S.K., Chung C.-K. (2004). Familiarity with, Knowledge of, and Attitudes toward Epilepsy in Residents of Seoul, South Korea. Acta Neurol. Scand..

[47] AlHarbi F.A., Alomari M.S., Ghaddaf A.A., Abdulhamid A.S., Alsharef J.F., Makkawi S. (2021). Public Awareness and Attitudes toward Epilepsy in Saudi Arabia: A Systematic Review and Meta-Analysis. Epilepsy Behav..

[48] Diamantopoulos N., Kaleyias J., Tzoufi M., Kotsalis C. (2006). A Survey of Public Awareness, Understanding, and Attitudes toward Epilepsy in Greece. Epilepsia.

[49] Jensen R., Dam M. (1992). Public Attitudes toward Epilepsy in Denmark. Epilepsia.

[50] Spatt J., Bauer G., Baumgartner C., Feucht M., Graf M., Mamoli B., Trinka E., Austrian Section of the International League Against Epilepsy (2005). Predictors for Negative Attitudes toward Subjects with Epilepsy: A Representative Survey in the General Public in Austria. Epilepsia.

[51] Gosavi T.D., Wang S., See S.J., Ng J., Lim S.H. (2018). Revisiting the Public Awareness, Attitudes, and Understanding towards Epilepsy among Singapore Residents. Epilepsy Behav..

[52] Jacob L., Kerimbaeva Z., Kalyapin A., Kostev K. (2019). Prescription Patterns of Antiepileptic Drugs in Kazakhstan in 2018: A Retrospective Study of 57,959 Patients. Epilepsy Behav..

[53] Lee J.W., Dworetzky B. (2010). Rational Polytherapy with Antiepileptic Drugs. Pharmaceuticals.

[54] Guekht A., Zharkinbekova N., Shpak A., Hauser W.A. (2017). Epilepsy and Treatment Gap in Urban and Rural Areas of the Southern Kazakhstan in Adults. Epilepsy Behav..

